# Mental Health Conditions and Incident Cancer: A Prospective Cohort Study of 402,255 UK Biobank Participants

**DOI:** 10.1002/ijc.70527

**Published:** 2026-04-28

**Authors:** Mohammed Sherif Amin, Solange Parra‐Soto, Ziyi Zhou, Shinya Nakada, Ike Dhiah Rochmawati, Carlos Celis‐Morales, Nancy Meligy, Jill P. Pell, Frederick K. Ho

**Affiliations:** ^1^ Department of Clinical Infection, Microbiology, and Immunology The University of Liverpool Liverpool UK; ^2^ School of Health and Wellbeing University of Glasgow Glasgow UK; ^3^ Department of Nutrition and Public Health, Faculty of Health Science and Food Universidad del Bío‐Bío Chillán Chile; ^4^ School of Cardiovascular and Metabolic Health University of Glasgow Glasgow UK; ^5^ Faculty of Pharmacy University of Surabaya Surabaya Indonesia; ^6^ Human Performance Lab, Education, Physical Activity and Health Research Unit University Católica del Maule Talca Chile; ^7^ Centro de Investigación en Medicina de Altura (CEIMA) Universidad Arturo Prat Iquique Chile; ^8^ Faculty of Medicine, Health and Life Sciences Swansea University Swansea UK

**Keywords:** anxiety, bipolar disorders, cancer epidemiology, depression, severe mental illness

## Abstract

Mental health conditions (MHCs) affect both psychological health and biological systems and have also been linked to cancer risk. However, evidence from epidemiological studies regarding this link remains inconsistent. We conducted a population‐based prospective cohort study involving 402,255 UK Biobank participants to investigate the associations of five MHCs (depressive disorders [DD], anxiety disorders [AD], bipolar disorder [BD], schizophrenia [SZ] and post‐traumatic stress disorder [PTSD]) with overall and site‐specific cancer risk. Cox proportional hazard models were used, adjusting for sociodemographic, health‐related and lifestyle confounders. Over a median follow‐up of 13.4 years, 68,065 (17%) incident cancer cases were recorded. DD (HR 1.18; 95% CI 1.13–1.23), AD (HR: 1.17, 95% CI: 1.11–1.24) and BD (HR: 1.29, 95% CI: 1.11–1.51) were associated with increased overall cancer risk. No significant association was found for SZ and PTSD. The associations of DD (HR: 1.27, 95% CI: 1.18–1.35) and BD (HR: 1.54, 95% CI: 1.26–1.88) were only significant in men. AD and DD were positively associated with lung, blood and liver cancers, while AD was also associated with prostate cancer. A dose–response relationship was observed between depressive symptom severity and cancer risk. While we cannot assume causality, our finding suggests that diagnoses of MHCs could be useful for cancer risk stratification and prevention.

## Abstract (highlights)

What's new?

Evidence suggests a potential association between mental health conditions and cancer risk, yet previous studies were limited by small samples and the use of self‐reported measures. Using data from the UK Biobank's large‐scale prospective population cohort over a median follow‐up of 13.4 years, this analysis demonstrates associations between depression, anxiety and bipolar disorders with overall and site‐specific cancers, with clear sex differences. A dose–response relationship was observed between depressive symptom severity and cancer risk. While causality cannot be assumed, diagnoses of mental health conditions could be useful for cancer risk stratification and prevention.

## Graphical abstract

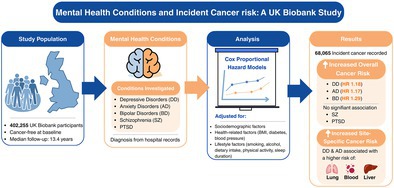

**Abbreviations**
95% CI: 95% confidence intervals
AD: anxiety disorders
BCSP: Breast Cancer Screening Programme
BD: bipolar disorders
BMI: body mass index
DD: depressive disorders
FIT: faecal immunochemical test
HR: hazard ratio
ICD: international classification of disease
IQR: interquartile ranges
kg/m^2^
: kilogram per meter squared
MET: total metabolic equivalent task
MHCs: mental health conditions
mmHg: millimeteres of mercury
PH: proportional hazard
PHQ‐4: 4‐item patient health questionnaire
PTSD: post‐traumatic stress disorder
SBP: systolic blood pressure
SD: standard deviations
SZ: schizophrenia
UKB: UK biobank

## 1 Introduction

The health burden from cancer is rising worldwide, with 20 million cases reported in 2022 [1]. Europe, in particular, faces a disproportionate impact, with the region contributing 22.4% of the global cancer cases while representing only 10% of the population [1]. Consistently, cancer incidence in the UK has risen by 0.8% annually over the past 25 years [2] despite the significant advancement in preventive initiatives targeting well‐established risk factors such as smoking [3] and alcohol consumption [4]. Notably, these factors only contribute to about 37% of cancer cases [3], highlighting the need to explore other prevalent modifiable risk factors to mitigate the escalating burden.

Mental health conditions (MHCs) have been previously linked to various physical conditions [5], especially cancer [6]. Traditionally, studies examined MHCs as a consequence of cancer. However, since Blumberg et al.'s [7] pioneering study in 1954 suggested a bidirectional relation, numerous studies have investigated the link between MHCs and cancer risk. Nevertheless, epidemiological evidence regarding this association is largely inconsistent [8]. Some studies reported a higher cancer risk [9, 10, 11], while others demonstrated either non‐significant associations [12] or even a lower risk for certain cancers [5, 12].

Although population‐based differences in key risk factors may contribute to this inconsistency, methodological limitations are likely more influential. For instance, the reliance on self‐reported data by most studies [9, 10, 11, 13] might introduce systematic bias. Driven by stigma, participants may tend to underreport socially undesirable conditions such as mental disorders in order to conform to perceived social norms, a phenomenon known as social desirability bias [14]. This issue is compounded by the fact that many studies adopt retrospective designs [15], which further introduce recall bias. A study comparing participants' ability to recall their depression symptoms [16] found that less than half of the participants recalled their key symptoms, and recall ability largely depends on the condition severity, a factor that is often overlooked by most studies.

In addition, many previous studies [11, 17, 18, 19] have been restricted by relatively small sample sizes, meaning that, even when data on various MHCs are available, studies have limited ability to perform stratified analyses across multiple mental disorders and cancer sites simultaneously due to reduced statistical power. A further limiting factor was the inadequate adjustment for confounding factors [8]. While crucial behavioural risk factors like smoking, alcohol consumption, physical activity levels and co‐morbidities such as diabetes and hypertension can significantly influence both MHCs [20] and cancer development [21, 22], the majority of studies [5, 11, 18, 23] did not account for these variables.

The current study aimed to address these limitations by using linked electronic health records and validated clinical assessments from the large prospective UK Biobank (UKB) cohort [24]. This approach reduces reliance on retrospective self‐reports and allows for temporal ordering of MHCs and cancer outcomes. Furthermore, the detailed data available in UKB enabled more comprehensive adjustment for confounding factors. We investigated the associations between five common MHCs (depressive disorders (DD), anxiety disorders (AD), bipolar disorder (BD), schizophrenia (SZ) and post‐traumatic stress disorder [PTSD]) with overall and site‐specific cancer incidence. Additionally, the dose–response relationship by depressive symptom severity and effect modifications by sex were also explored.

## 2 Materials and Methods

### 2.1 Study Design

The UKB is a population‐based cohort that recruited 502,493 participants between 2006 and 2010 across England, Wales and Scotland [24]. Participants provided comprehensive data through self‐administered touch screen questionnaires and in‐person assessments, covering various demographics, behavioural habits, medical history and physical measurements. Health outcomes were tracked through linkage with UK administrative data, including hospital records and death registries [24]. A detailed description of the UKB is available at https://www.ukbiobank.ac.uk.

### 2.2 MHCs

MHCs were ascertained through a confirmed diagnosis of DD, AD, BD, SZ and PTSD identified through linked inpatient hospital admission records. Records were available until March 2021 for data from England and Scotland, while Wales' data were available till February 2018. In the UKB, condition diagnosis was coded using ICD‐10; therefore, participants diagnosed under the following codes were included in the study: F32/F33 for DD, F40–F43 for AD, F31 for BD, F20 for SZ and F43.1 for PTSD.

**FIGURE 1:**
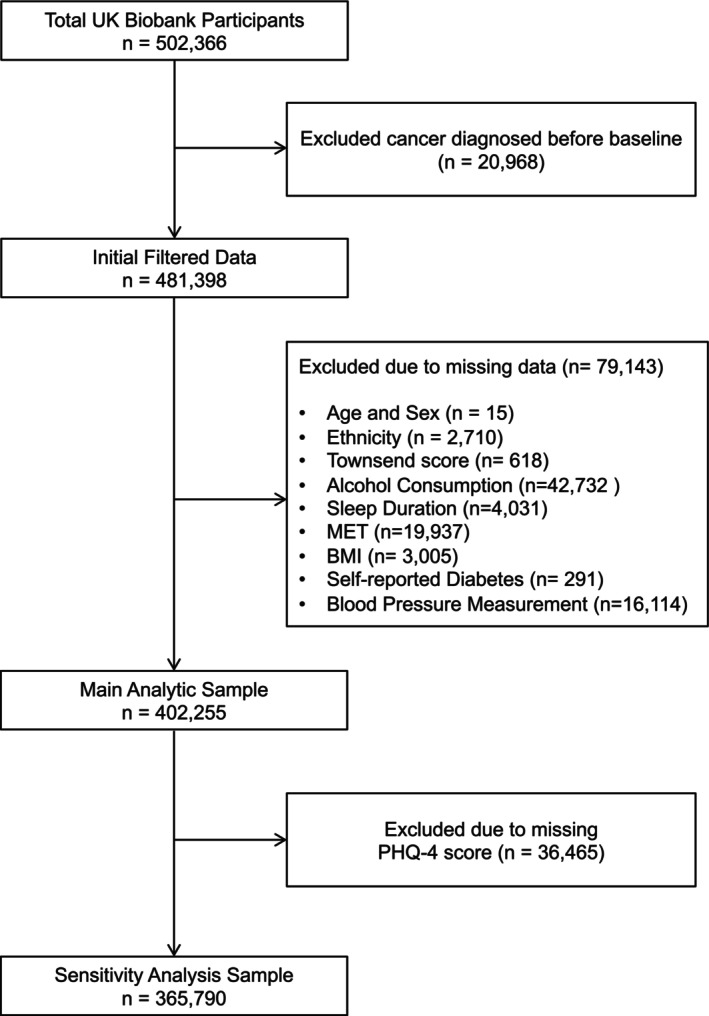
Flow diagram of the study sample selection. AD, anxiety disorders; BD, bipolar disorders; DD, depressive disorders; MHCs, mental health conditions; PHQ‐4, 4‐item patient health questionnaire; PTSD, post‐trumatic stress disorders; SZ, shizophrenia.

Participants diagnosed with each disorder at or before the baseline assessment (between 2006 and 2010) were classified as ‘diagnosed’ to that specific condition, with the rest of the UKB participants serving as the comparison group. Diagnoses made after the baseline but before any cancer diagnosis were also classified as ‘diagnosed’, after which the follow‐up started. Figure 1 shows a flow diagram of the selection process.

DD and AD symptom severity were assessed using the 4‐item patient health questionnaire (PHQ‐4) collected at baseline. Each question is scored from 0 (‘not at all’) to 3 (‘nearly every day’), and the total score ranges from 0 (no symptoms) to 12 (severe symptoms) [25].

### 2.3 Cancer Outcomes

To maintain a cancer‐free baseline, those with a pre‐baseline cancer diagnosis (*n* = 20,968) were excluded. Overall and site‐specific incident cancers were identified as the first eligible code in either inpatient and day‐case hospital records or death records.

Accordingly, the ‘overall cancer’ variable represented the post‐baseline incident cases under ICD‐10 codes C00–C39, C43 and C50–C97 (i.e., all cancers except non‐melanotic skin and bone cancers). For site‐specific analyses, the study analysed eight common types of cancer: breast (C50), ovarian (C56), uterine (C54–C55), prostate (C61), lung (C33–C34), blood (C81–C96), colorectal (C18–C20) and liver (C22).

### 2.4 Covariates

Covariates were selected based on prior evidence of potential confounding or mediating effects [8], including sociodemographic, health‐related and behavioural/lifestyle factors retrieved from the UKB's baseline assessment.

#### 2.4.1 Sociodemographic Factors

Age, sex and ethnicity were self‐reported. Townsend area deprivation index was obtained from the postcode of residence and is derived using aggregated data on unemployment, car and home ownership and household overcrowding [26].

#### 2.4.2 Health‐Related Factors

Body Mass Index (BMI) was calculated based on participants' weight and height (kilograms per meter squared (kg/m^2^)). Diabetes was self‐reported based on a physician's diagnosis as ‘yes’ or ‘no’. Systolic blood pressure (SBP) was measured either using an automated device or a sphygmomanometer and reported in millimetres of mercury (mmHg). Lastly, female menopausal status (‘Yes’ or ‘No’) was considered, as hormonal changes during menopause can influence mood disorders like DD and ad [27] and increase the risk of certain cancers, such as breast and uterine cancer [28].

#### 2.4.3 Behavioural/Lifestyle Factors

Smoking was self‐reported as ‘Current’, ‘Previous’ or ‘Never’, with any other category re‐coded as missing in the study. Alcohol consumption was self‐reported as units per week (1 unit = 10 mL of ethanol). Physical activity was self‐reported using the International Physical Activity Questionnaire Short Form as total Metabolic Equivalent Task (MET). Sleep duration was self‐reported based on hours of sleep over a 24‐h period. Lastly, processed meat consumption was self‐reported as the average intake over the past year, coded as ‘Never’, ‘Less than once a week’, ‘Once a week’, ‘2–5 times a week’, ‘5–6 times a week’, ‘once or more daily’.

### 2.5 Statistical Analysis

Descriptive statistics established baseline characteristics, stratified by sex and MHC diagnosis, using means (standard deviation [SDs]) for normally distributed continuous variables, medians (Interquartile Range [IQR]) for skewed data and frequencies and percentages for categorical variables.

The association between MHCs and overall and site‐specific cancer incidence was examined using Cox proportional hazard (PH) models, producing Hazard Ratios (HR) and corresponding 95% Confidence Intervals (95% CI). Participants with missing data for included covariates were excluded from the analysis (*n* = 79,143). The dependent variable was the time until cancer occurrence, calculated as the years from the first MHC diagnosis to the cancer diagnosis, death or study end, whichever came first. All analyses were conducted using a 1‐year landmark to reduce the potential of reverse causality. This ensures that mental health symptoms are not merely a reaction to an undiagnosed early‐stage tumour, but rather that the MHC preceded the cancer diagnosis.

Three incrementally adjusted Cox models were used to explore the potential pathways through which MHCs influence cancer risk. Model 1 adjusted for sociodemographic factors, Model 2 additionally adjusted for health‐related factors and Model 3 further included lifestyle/behavioural factors. For female‐specific cancers (e.g., breast, ovarian, uterine), Model 2 was also adjusted for menopausal status. This specific sequence was adopted to delineate the association beyond the baseline comorbidities and to suggest the extent to which behavioural factors might play a mediating role in these associations. PH assumptions were assessed using Schoenfeld residuals.

Initially, models were conducted for each MHC and overall cancer. Site‐specific cancers were only analysed if there was a minimum of five cases per predictor variable to ensure reliability. Interaction terms were included in the fully adjusted models (Model 3) to examine the sex‐moderating effect, with further sex stratification for conditions showing significant interactions.

In addition to clinically recorded diagnoses, standardised PHQ‐4 scores were included as an exposure in a sensitivity analysis to capture subclinical cases and to examine potential dose–response relationships of symptom severity on the association. This approach allowed for a more complete assessment of the mental health burden beyond binary diagnostic categories. Furthermore, we calculated *E*‐values [29] to quantify the potential impact of unmeasured confounding on our results. *E*‐values determine the minimum association strength required for an uncontrolled variable to move the observed HRs to the null, with higher values suggesting that residual confounding is likely minimal [29]. All analyses were performed using R version 4.3.1 and set at a significance level of alpha = 0.05.

## 3 Results

Of the 402,255 UK Biobank participants, 39,304 (9.8%) MHCs cases were identified (DD: 25,616 [6.4%], AD: 21,892 [5.4%], BD: 1510 [0.4%], SZ: 1719 [0.4%], PTSD: 477 [0.1%]). Over a median follow‐up of 13.4 (IQR: 12.6, 14.3) years, 68,065 (17%) incident cancer cases were observed. Site‐specific cancer incidence is detailed in Table S1.

Overall, participants were mostly of white ethnicity (95%) and generally healthy in terms of lifestyle factors. The mean alcohol consumption was 11 units/week and over half (55%) reported never smoking (Table 1). Diabetes prevalence was low (4.5%). The mean SBP was elevated (138 mmHg), placing participants within the ‘elevated blood pressure’ category according to the European Society of Cardiology (ESC) guidelines [30].

**TABLE 1:** Baseline characteristics of included participants (*n* = 402,255) stratified by MHC diagnosis.

Characteristic	Overall *N* = 402,255	Undiagnosed *N* = 362,951	Diagnosed *N* = 39,304	*p* ^d^
Age^a^ (years)	56 (8)	56 (8)	57 (8)	< 0.001
sex^b^				< 0.001
Female	214,015 (53%)	189,493 (52%)	24,522 (62%)	
Male	188,240 (47%)	173,458 (48%)	14,782 (38%)	
Ethnicity^b^				< 0.001
White	380,847 (95%)	343,419 (95%)	37,428 (95%)	
Black	6235 (1.6%)	5714 (1.6%)	521 (1.3%)	
Chinese	1277 (0.3%)	1226 (0.3%)	51 (0.1%)	
Mixed	2360 (0.6%)	2062 (0.6%)	298 (0.8%)	
South Asian	7975 (2.0%)	7314 (2.0%)	661 (1.7%)	
Any other	3561 (0.9%)	3216 (0.9%)	345 (0.9%)	
Deprivation^a^	−1.37 (3.04)	−1.45 (3.00)	−0.69 (3.34)	< 0.001
Smoking status^b^				< 0.001
Current	41,452 (10%)	34,910 (9.6%)	6542 (17%)	
Never	219,829 (55%)	201,336 (55%)	18,493 (47%)	
Previous	140,974 (35%)	126,705 (35%)	14,269 (36%)	
Alcohol^c^ (units/week)	11 (3, 23)	11 (3, 23)	8 (2, 21)	< 0.001
Sleep Duration^a^ (hour)	7.16 (1.08)	7.15 (1.04)	7.17 (1.37)	0.004
Total physical activity (MET‐min/week)^a^	2422 (2439)	2438 (2432)	2281 (2501)	< 0.001
Processed meat consumption^a^				< 0.001
Never	37,547 (9.3%)	33,384 (9.2%)	4163 (11%)	
Less than once a week	121,657 (30%)	110,004 (30%)	11,653 (30%)	
Once a week	117,335 (29%)	106,214 (29%)	11,121 (28%)	
2–5 times a week	109,667 (27%)	99,012 (27%)	10,655 (27%)	
5–6 times a week	12,787 (3.2%)	11,468 (3.2%)	1319 (3.4%)	
once or more daily	3262 (0.8%)	2869 (0.8%)	393 (1.0%)	
BMI^a^ (kg/m^2^)	27.3 (4.7)	27.2 (4.6)	28.3 (5.5)	< 0.001
Self‐reported Diabetes^b^	18,006 (4.5%)	15,341 (4.2%)	2665 (6.8%)	< 0.001
SBP^a^ (mmHg)	138 (19)	138 (19)	137 (19)	< 0.001
Menopausal status^b^				< 0.001
No	52,377 (13%)	47,260 (13%)	5117 (13%)	
Yes	128,630 (32%)	114,092 (31%)	14,538 (37%)	
Not applicable/unknown	221,248 (55%)	201,599 (56%)	19,649 (50%)	
Follow‐up time^c^ (year)	13.4 (12.6, 14.3)	13.4 (12.6, 14.3)	13.2 (11.9, 14.1)	< 0.001

*Note:* Significance level set at alpha = 0.05.

Abbreviations: BMI, body mass index; MET, metabolic equivalent task; SBP, systolic blood pressure.

^a^ Mean, (SD).

^b^ *n* (%).

^c^ Median (IQR).

^d^ Welch two sample *t*‐test; Pearson's Chi‐squared test; Wilcoxon rank sum test.

Table 1 presents baseline characteristics stratified by MHC status. Participants with MHCs exhibited poorer health profiles compared to those without MHCs, including higher smoking (diagnosed: 17%, undiagnosed: 9.6%) and diabetes prevalence (diagnosed: 6.8%, undiagnosed: 4.2%) and lower MET scores. Notably, in the sex‐stratified characteristics (Table S2), men demonstrated higher mean BMI and SBP, and smoking, alcohol consumption, and diabetes rates than women.

**FIGURE 2:**
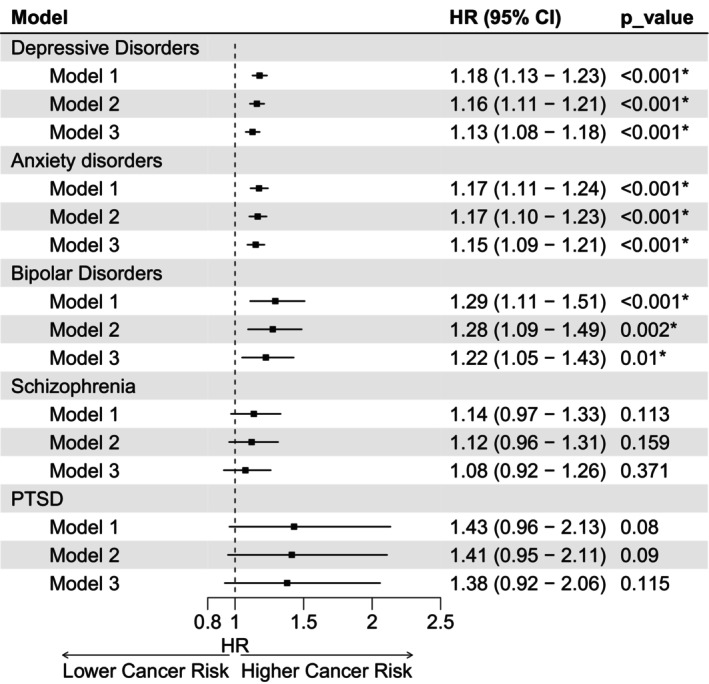
Forest plot of the associations between MHCs and overall cancer incidence (*n* = 402,255). CI, confidence Interval; HR, hazard ratio; PTSD, post‐traumatic stress disorders. Model 1 adjusted for sociodemographic factors; Model 2 adjusted for sociodemographic and health‐related factors; Model 3 adjusted for sociodemographic factors, health‐related, and lifestyle factors. *Significance level set at alpha = 0.05.

Associations between MHCs and overall cancer incidence are presented in Figure 2. In Model 1, three of the five MHCs investigated were associated with overall cancer risk: DD (HR: 1.18, 95% CI: 1.13–1.23), AD (HR: 1.17, 95% CI: 1.11–1.24) and BD (HR: 1.29, 95% CI: 1.11–1.51). Although slightly attenuated, these associations remained statistically significant in Model 3, with HRs of 1.13 (1.08–1.18) for DD, 1.15 (1.09–1.21) for AD and 1.22 (1.05–1.43) for BD. Meanwhile, no significant association was found for SZ and PTSD across the three models. The PH assumption was met for all models except for the DD fully adjusted model (*p*‐value = 0.01).

Interaction terms between each MHC and sex were tested using likelihood ratio tests. Only the interactions for DD and BD were statistically significant (Table S3), thus both were further stratified by sex. The sex‐stratified analysis only found significant associations of DD (HR: 1.27, 95% CI: 1.18–1.35) and BD (HR: 1.54, 95% CI: 1.26–1.88) in men (Figure S1).

**FIGURE 3:**
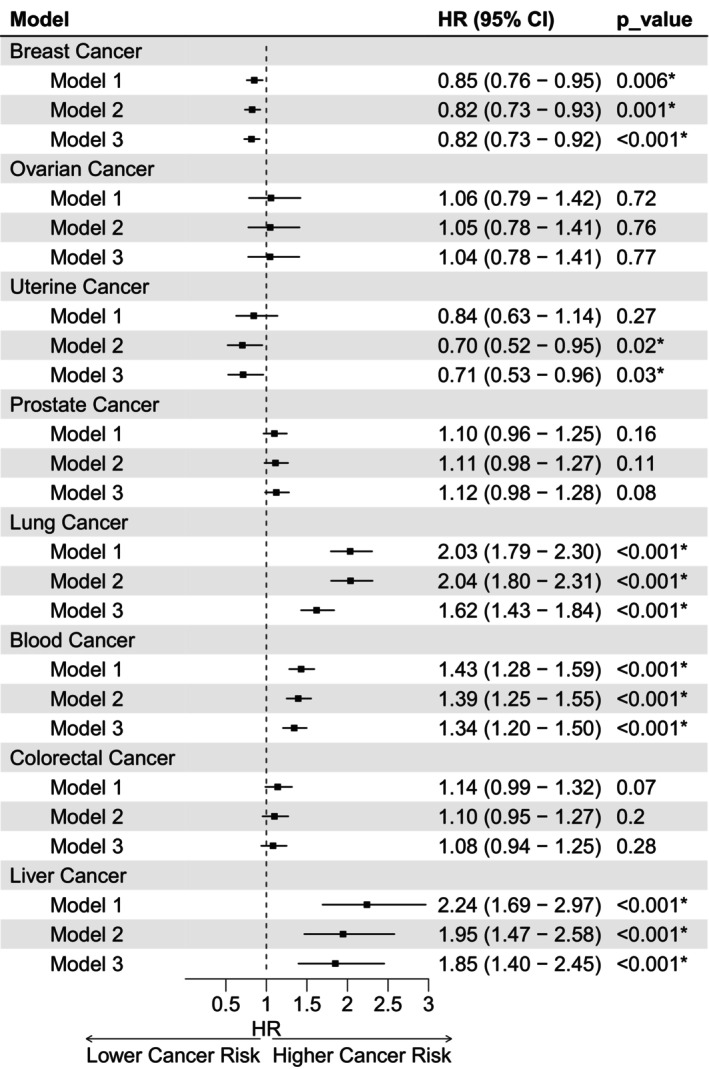
Forest plot for Cox models of depressive disorders and site‐specific cancer incidence. CI, confidence Interval; HR, hazard ratio; PTSD, post‐traumatic stress disorders. Model 1 adjusted for sociodemographic factors; Model 2 adjusted for sociodemographic and health‐related factors; Model 3 adjusted for sociodemographic factors, health‐related, and lifestyle factors. *Significance level set at alpha = 0.05.

**FIGURE 4:**
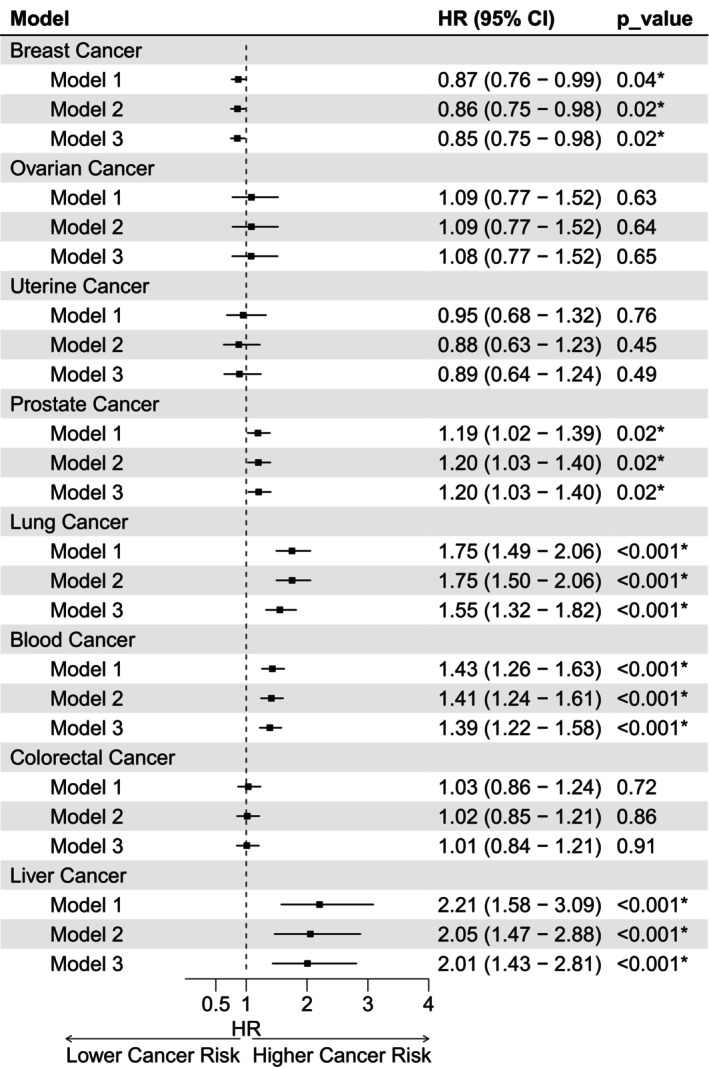
Forest plot for Cox models of anxiety disorders and site‐specific cancer incidence. CI, confidence Interval; HR, hazard ratio; PTSD, post‐traumatic stress disorders. Model 1 adjusted for sociodemographic factors; Model 2 adjusted for sociodemographic and health‐related factors; Model 3 adjusted for sociodemographic factors, health‐related, and lifestyle factors. *Significance level set at alpha = 0.05.

For site‐specific cancers, all three models for DD (Figure 3) and AD (Figure 4) were positively associated with lung, blood and liver cancers, while models for AD were also associated with prostate cancer. Notably, adjustment for health‐related factors in Model 2 resulted in minimal attenuation of the HRs for most cancer outcomes, whereas a more pronounced attenuation was observed after the inclusion of behavioural factors in Model 3. For DD, the HR for lung cancer was 2.04 (95% CI: 1.80–2.31) in Model 2, which attenuated to 1.62 (95% CI: 1.43–1.84) in Model 3. A similar pattern was observed for AD (Model 2: HR 1.75, 95% CI: 1.50–2.06; Model 3: HR 1.55, 95% CI: 1.32–1.82).

Contrastingly, DD and AD were associated with a reduced risk of breast cancer, showing 18% and 15% reduction for Model 3, respectively. The association for BD, SZ and PTSD could not be modelled due to insufficient statistical power.

After excluding 36,465 individuals with missing PHQ‐4 scores, the sensitivity analysis was conducted on 365,790 participants. A positive dose–response relationship was observed between PHQ‐4 score and overall cancer risk [HR: 1.03 (95% CI: 1.02–1.04)] as well as prostate, lung, blood and liver cancer risk (Figure S2). Higher PHQ‐4 symptom scores were also associated with increased breast cancer risk, although this did not reach statistical significance.

Table S4 presents the *E*‐values calculated based on Model 3 estimates. *E*‐values for overall cancer risk ranged from 1.36 to 2.10. For the associations between DD and site‐specific cancers, *E*‐values ranged from 1.26 to 3.10, with the highest observed for liver and lung cancers. Similarly, for AD, site‐specific *E*‐values ranged from 1.11 to 3.43.

## 4 Discussion

### 4.1 Main Findings

This largescale prospective population cohort study demonstrated significant associations between DD, AD and BD and the overall risk of subsequently being diagnosed with cancer, as well as the risk of a number of site‐specific cancers. These associations persisted after adjustment for sociodemographic, health‐related and behavioural factors, although the degree of attenuation varied by cancer site. Interestingly, a protective association was observed for breast cancer in women, which requires further investigation. The lack of significant associations with SZ and PTSD should be corroborated in other studies or meta‐analyses due to their lower statistical power.

The number of MHCs and outcomes implicated suggests possible common pathways operating across a range of conditions. Prior research supports this notion, highlighting a potential genetic overlap between MHCs, with a Brazilian study [31] identifying a 66% genetic overlap between depression and anxiety symptoms. One hypothesis is that this shared genetic component may also contribute to increased cancer risk, possibly through pleiotropic effects (i.e., genes influencing multiple traits); however, this remains speculative and requires further investigation in future studies.

### 4.2 Sex Differences

Sex‐stratified analyses revealed a male‐specific association for DD and BD with overall cancer risk. This inequality likely reflects sex‐based behavioural differences as men tend to engage more frequently in behaviours that are recognised risk factors for both MHCs and cancer, such as smoking and alcohol consumption. Even though we adjusted for these factors, there could be residual confounding. The UK's Office for National Statistics 2022 report [32] and the 2021 Health Survey for England [33] highlight these differences, showing higher smoking (14.6%) and alcohol consumption (32%) prevalence among men compared to women (11.2%, 12%, respectively). Additionally, lower healthcare‐seeking behaviour among men with MHCs may have also contributed to this inequality. In the UK, between 2020 and 2021, the number of men (*n* = 469,756) referred to the Improving Access to Psychological Therapies (IAPT) programme, designed to address depression and anxiety, was almost half that of women (*n* = 975,399) [34]. This underrepresentation could lead to delayed diagnoses and potentially more advanced disease stages in men, influencing the observed association with overall cancer risk.

### 4.3 Site‐Specific Cancer Risk

Regarding site‐specific cancer risk, DD and AD were associated with higher risks of several cancers, yet displayed a potential protective effect against breast cancer. Our findings are consistent with previous studies [9, 35] and may reflect differential uptake of breast cancer screening programmes rather than a true biological protection. Since 1988, UK women aged 50–70 years have been invited to the Breast Cancer Screening Programme (BCSP) every 3 years [36]. However, the programme relies on voluntary participation and studies show that women with MHCs have 15% lower odds of attending screenings [37]. Given our sample's eligibility for screening, this lower participation rate might explain the observed lower breast cancer risk associated with MHC diagnosis in our group.

Notably, this mechanism of ‘apparent protection’ may not apply to other screened cancers in the same way. For example, many colorectal cancers are mostly diagnosed following symptomatic presentation rather than through screening alone [38]. Consequently, lower screening uptake among individuals with MHCs for other sites does not necessarily mask the increased risk to the same extent as it does for breast cancer, where asymptomatic detection is a primary driver of recorded incidence. This interpretation is further supported by the discrepancy observed in our sensitivity analysis. While clinically diagnosed DD and AD appeared protective for breast cancer, higher PHQ‐4 symptom scores trended towards increased breast cancer risk. One possible explanation is that diagnostic records may capture individuals whose observed cancer incidence is influenced by differential screening participation and healthcare access, whereas the underlying psychological distress itself may still align with the expected positive association between MHCs and cancer risk. Future research should compare breast cancer stage at diagnosis between those with and without MHCs to confirm if these ‘protected’ individuals are simply being diagnosed later.

### 4.4 Comparison With Previous Research

Previous research predominantly focused on the association between depression or anxiety and overall cancer risk [11, 13, 17, 23, 39], likely reflecting their higher prevalence worldwide. A consistent pattern emerged among most studies demonstrating an elevated cancer risk among individuals with these conditions [9, 11, 17, 18]. Nonetheless, a UK investigation based on the Whitehall II study [39] reported no significant association between depression and overall cancer risk. Although this finding seemingly contradicts our observation, several methodological limitations could have impacted their findings. The study utilised historical NHS records from 1991 to detect cancer incidence, which were recognised as very incomplete, resulting in 34% missing data in the study [40]. Additionally, the study had much lower statistical power than ours, with a sample size of 6983 and 776 incident cancer cases, compared with 402,255 and 68,065, respectively, in our study.

Few studies have examined the associations with different cancer sites, with lung cancer being the most studied. The current study aligns with prior evidence showing a higher risk of lung cancer associated with depression [10, 23] and anxiety [41]. Evidence linking MHCs to other cancers is even scarcer. To our knowledge, only one study [42] investigated the association between depression and liver cancer risk and reported no association (OR: 0.81, 95% CI: 0.59–1.11). However, this finding was based on a retrospective investigation of 114 participants. In contrast, our study identified liver cancer as one of the strongest site‐specific associations.

This strong association may reflect the convergence of multiple pathways. First, behavioural pathways, where chronic alcohol consumption is a major risk factor for liver cancer and is frequently utilised as a maladaptive coping mechanism in individuals with MHCs [43]. While we adjusted for a comprehensive alcohol intake variable, it was reported that heavy drinkers tend to be underreported. This leads to residual confounding, or the cumulative duration of heavy drinking may contribute to this strong effect. Furthermore, the liver is highly sensitive to metabolic dysfunction; MHCs are associated with systemic inflammation [44] and the development of non‐alcoholic fatty liver disease [45], which are recognised precursors to hepatocellular carcinoma. The intersection of these behavioural and metabolic pathways likely accounts for the pronounced liver cancer risk observed in this study.

### 4.5 Potential Mechanisms

The variation in associations across different cancer sites suggests that the influence of MHCs is not uniform but operates through site‐specific mechanisms. Our findings of strong associations with lung and liver cancers align with a behavioural pathway, where MHCs act as distal drivers of tobacco and alcohol use. In contrast, the associations observed with blood malignancies, which are less tied to these specific lifestyle habits, point towards a possible biological pathway.

Meanwhile, the null or protective findings for sites like breast cancer highlight the role of healthcare‐seeking behaviour and detection bias. This thematic divergence, where some sites reflect lifestyle, others biology and others the limitations of screening, explains why MHCs do not show a universal association across all cancers. This nuance is consistent with wide evidence suggesting that the ‘psychosocial‐cancer’ link is highly dependent on the specific aetiology of the malignancy in question [8].

### 4.6 Strengths and Limitations

Our study had a number of strengths. First, the large UK Biobank sample ensured sufficient power to examine associations across five MHCs and eight cancer sites, as well as testing of interactions with sex and subgroup analyses. Second, utilising administrative data to ascertain outcomes minimised the risk of information bias. Third, the study involved a comprehensive set of covariates, allowing for the adjustment of most recognised confounders. Last, the study's long follow‐up provided a sufficient observation period for cancer development, as a previous systematic review [46] showed that studies with less than 10‐year follow‐up were less likely to detect significant associations.

However, the study has limitations. As an observational study, it cannot establish causation. Despite thorough covariate adjustment, residual or unmeasured confounding cannot be ruled out. Nonetheless, our sensitivity analysis using *E*‐values provides a quantitative measure of reassurance regarding the stability of our estimates. The relatively high *E*‐values for site‐specific risks, particularly lung (DD: 2.62, AD: 2.48) and liver cancer (DD: 3.10, AD: 3.43), suggest that it is unlikely an unmeasured factor exists with sufficient explanatory power to fully account for these associations.

A further limitation is the use of a complete case approach, which excluded 79,143 participants with missing covariate data. This approach was adopted to ensure consistency across the three incrementally adjusted models by restricting analyses to participants with complete data on all covariates included in the fully adjusted model (Model 3). To assess the potential impact of this decision, we compared participants included in the final analytic sample with those excluded due to missing data (Table S5). Excluded participants tended to be female, from minority ethnic groups, more deprived, current smokers, with lower physical activity, higher BMI, diabetes and a recorded MHC diagnosis, suggesting that missingness was not entirely random. As these characteristics are associated with both MHCs and cancer risk, exclusion may have introduced selection bias.

The direction of this bias is difficult to determine; however, excluding participants with a less favourable risk profile may have led to underestimation of the true associations. Alcohol consumption and SBP were among the variables contributing to missingness and are particularly relevant. Alcohol may act as both a confounder and mediator, especially for liver [47] and colorectal cancers [48], while SBP may reflect broader cardiometabolic risk. Nonetheless, given that no variable exceeded 10% missingness, we do not expect our findings to change with alternative analytic strategies, such as multiple imputation.

MHC diagnoses in our study were not mutually exclusive. Participants were classified as having a given condition if they had a recorded diagnosis for that disorder, regardless of co‐occurrence with other conditions. This may have inflated the estimated risk for individuals with multiple co‐occurring conditions. As such, these estimates likely reflect the cumulative burden of mental health comorbidity rather than isolated effects.

Furthermore, the prevalence of MHCs at baseline included self‐report of physician diagnoses, but MHCs diagnosed thereafter were ascertained from hospital records, potentially excluding less severe cases managed exclusively in the community. Nonetheless, the observed median PHQ‐4 score of 2 within the sample suggests that the analysis captured a range of MHC severities, including less severe cases. The reliance on hospital admissions or deaths to ascertain cancer outcomes may have resulted in incomplete ascertainment of early and less severe cases managed in the community or outpatients; however, this would mainly apply to skin cancers, which were not included in the study.

The PH assumption violations in model 3 of DD‐overall cancer, DD‐breast cancer, AD‐breast, AD‐ovarian and AD‐colorectal cancer may affect HR interpretation. For these models, HRs should be interpreted as a weighted average effect over the study period, as suggested in a recent study [49]. Lastly, the UK Biobank's low baseline response rate (5.5%) raises concerns about its representativeness. However, previous studies [50] showed that associations obtained from the UK Biobank were broadly consistent with those from more representative studies, supporting their generalisability.

In conclusion, a range of MHCs (DD, AD and BD) were associated with a higher risk of cancer overall and several site‐specific cancers. While causality cannot be assumed, diagnoses of MHCs could be useful for cancer risk stratification, as these individuals might represent a high‐risk group. Furthermore, the findings reinforce the need to support healthy lifestyles among people with MHCs.

## Author Contributions

**Mohammed Sherif Amin:** conceptualization, formal analysis, visualization, writing – original draft, methodology, writing – review and editing. **Solange Parra‐Soto:** writing – review and editing. **Ziyi Zhou:** writing – review and editing. **Shinya Nakada:** writing – review and editing. **Ike Dhiah Rochmawati:** writing – review and editing. **Carlos Celis‐Morales:** writing – review and editing. **Nancy Meligy:** writing – review and editing. **Jill P. Pell:** writing – review and editing, funding acquisition. **Frederick K. Ho:** conceptualization, methodology, writing – review and editing, supervision.

## Ethics Statement

The authors assert that all procedures contributing to this work comply with the ethical standards of the relevant national and institutional committees on human experimentation and with the Helsinki Declaration of 1975, as revised in 2013. All procedures involving human subjects were approved under UK Biobank's generic ethical approval (Ref: 11/NW/0382), and this study is registered as UK Biobank project number 71392.

## Conflicts of Interest

The authors declare no conflicts of interest.

## Supporting information


**Table S1:** Summary statistics of cancer incidence stratified by MHCs diagnosis.
**Table S2:** Baseline characteristics of included participants stratified by sex (*n* = 402,255).
**Table S3:** Likelihood Ratio Tests comparing Cox models with and without interaction terms between MHCs and sex in relation to cancer incidence.
**Table S4:** Sensitivity analysis for unmeasured confounding showing the hazard ratios and corresponding *E*‐Values for MHCs and cancer risk.
**Table S5:** Baseline characteristics of participants included in the analytic sample (*n* = 402,255) and those excluded due to missing covariate data (*n* = 79,143).
**Figure S1:** Forest plot of Cox models for depressive disorders and bipolar disorders and overall cancer stratified by sex.
**Figure S2:** Forest plot of the sensitivity analysis models using PHQ‐4.

## Data Availability

This work has been conducted using the UK Biobank Resource under application 71,392. The UK Biobank is an open access resource and bona fide researchers can apply to use the UK Biobank dataset by registering and applying at http://ukbiobank.ac.uk/register‐apply/. Further information is available from the corresponding author upon request.
