# Supplementary material for: Mental Health Conditions and Incident Cancer: A Prospective Cohort Study of 402,255 UK Biobank Participants
Source: Int J Cancer. 2026 Apr 28;159(6):1420–31. doi: 10.1002/ijc.70527 (PMC13397219; doi:10.1002/ijc.70527)
Supplement: Supplementary file 1 — Table S1: Summary statistics of cancer incidence stratified by MHCs diagnosis. Table S2: Baseline characteristics of included participants stratified by sex (n = 402,255). Table S3: Likelihood Ratio Tests comparing Cox models with and without interaction terms between MHCs and sex in relation to cancer incidence. Table S4: Sensitivity analysis for unmeasured confounding showing the hazard ratios and corresponding E‐Values for MHCs and cancer risk. Table S5: Baseline characteristics of participants included in the analytic sample (n = 402,255) and those excluded due to missing covariate data (n = 79,143). Figure S1: Forest plot of Cox models for depressive disorders and bipolar disorders and overall cancer stratified by sex. Figure S2: Forest plot of the sensitivity analysis models using PHQ‐4. [file IJC-159-1420-s001.pdf]

Mental health conditions and incident cancer: a prospective cohort study of 402,255 UK Biobank participants

Mohammed Sherif Amin, Solange Parra-Soto, Ziyi Zhou, Shinya Nakada, Ike Dhiah Rochmawati, Carlos Celis-Morales, Nancy Meligy, Jill P Pell, Frederick K Ho

Table of Content

SUPPLEMENTARY TABLES 2

Supplementary Table 1 Summary statistics of cancer incidence stratified by MHCs diagnosis 2

Supplementary Table 2 Baseline characteristics of included participants stratified by sex (n = 402,255) 3

Supplementary Table 3 Likelihood Ratio Tests comparing Cox models with and without interaction terms between MHCs and sex in relation to cancer incidence 4

Supplementary Table 4 Sensitivity analysis for unmeasured confounding showing the hazard ratios and corresponding E-Values for MHCs and cancer risk 5

Supplementary Table 5 Baseline characteristics of participants included in the analytic sample (n=402,255) and those excluded due to missing covariate data (n=79,143) 6

SUPPLEMENTARY FIGURES 7

Supplementary Figure 1 Forest plot of Cox models for depressive disorders and bipolar disorders and overall cancer stratified by sex 7

Supplementary Figure 2 Forest plot of the sensitivity analysis models using PHQ-4 8

Supplementary tables

Supplementary Table 1 Summary statistics of cancer incidence stratified by MHCs diagnosis

Cancer Incidence[1]	All MHCs<br>N = 39,304	DD<br>N = 25,616	AD<br>N = 21,892	BD<br>N = 1,510	SZ<br>N = 1,719	PTSD<br>N = 477
Overall	9,122 (23%)	5,839 (23%)	5,303 (24%)	309 (20%)	358 (21%)	86 (18%)
Site-specific Cancer						
Breast	1,679 (4.3%)	1,043 (4.1%)	1,061 (4.8%)	51 (3.4%)	39 (2.3%)	15 (3.1%)
Ovarian	239 (0.6%)	150 (0.6%)	139 (0.6%)	10 (0.7%)	11 (0.6%)	3 (0.6%)
Uterine	271 (0.7%)	169 (0.7%)	169 (0.8%)	10 (0.7%)	7 (0.4%)	1 (0.2%)
Prostate	1,143 (2.9%)	739 (2.9%)	615 (2.8%)	53 (3.5%)	51 (3.0%)	15 (3.1%)
Lung	874 (2.2%)	579 (2.3%)	481 (2.2%)	30 (2.0%)	44 (2.6%)	5 (1.0%)
Blood	1,463 (3.7%)	907 (3.5%)	859 (3.9%)	49 (3.2%)	69 (4.0%)	14 (2.9%)
Colorectal	873 (2.2%)	547 (2.1%)	516 (2.4%)	27 (1.8%)	36 (2.1%)	10 (2.1%)
Liver	144 (0.4%)	96 (0.4%)	75 (0.3%)	4 (0.3%)	13 (0.8%)	2 (0.4%)

[1]n (%); MHCs: mental health conditions; DD: depressive disorders; AD: anxiety disorders; BD: bipolar disorders; SZ: schizophrenia; PTSD: post-traumatic stress disorders

Supplementary Table 2 Baseline characteristics of included participants stratified by sex (n = 402,255)

Characteristic	Overall<br>N = 402,255	Female<br>N = 214,015	Male<br>N = 188,240	p-value[4]
Age[1] (years)	56, (8)	56, (8)	57, (8)	<0.001
Ethnicity[2]				<0.001
White	380,847 (95%)	202,326 (95%)	178,521 (95%)	
Black	6,235 (1.6%)	3,624 (1.7%)	2,611 (1.4%)	
Chinese	1,277 (0.3%)	810 (0.4%)	467 (0.2%)	
Mixed	2,360 (0.6%)	1,470 (0.7%)	890 (0.5%)	
South Asian	7,975 (2.0%)	3,764 (1.8%)	4,211 (2.2%)	
Any other	3,561 (0.9%)	2,021 (0.9%)	1,540 (0.8%)	
Deprivation[1]	-1.37 (3.04)	-1.40 (2.99)	-1.35 (3.09)	<0.001
Smoking Status[2]				<0.001
Current	41,452 (10%)	18,601 (8.7%)	22,851 (12%)	
Never	219,829 (55%)	127,444 (60%)	92,385 (49%)	
Previous	140,974 (35%)	67,970 (32%)	73,004 (39%)	
Alcohol[3] (units/week)	11 (3, 23)	8 (2, 15)	18 (8, 33)	<0.001
Sleep Duration[1] (hour)	7.16 (1.08)	7.18 (1.09)	7.13 (1.06)	<0.001
Total physical activity (MET-min/wk)[1]	2,422 (2,439)	2,267 (2,282)	2,599 (2,595)	<0.001
Processed Meat Consumption[1]				<0.001
Never	37,547 (9.3%)	27,319 (13%)	10,228 (5.4%)	
Less than once a week	121,657 (30%)	81,519 (38%)	40,138 (21%)	
Once a week	117,335 (29%)	61,335 (29%)	56,000 (30%)	
2-5 times a week	109,667 (27%)	39,962 (19%)	69,705 (37%)	
5-6 times a week	12,787 (3.2%)	3,102 (1.4%)	9,685 (5.1%)	
once or more daily	3,262 (0.8%)	778 (0.4%)	2,484 (1.3%)	
BMI[1] (kg/m^2)	27.3 (4.7)	26.9 (5.1)	27.8 (4.2)	<0.001
Self-reported Diabetes[2]	18,006 (4.5%)	6,483 (3.0%)	11,523 (6.1%)	<0.001
SBP (mmHg)	138 (19)	135 (19)	141 (17)	<0.001
Menopausal Status[2]				<0.001
no	52,377 (13%)	52,377 (24%)	0 (0%)	
yes	128,630 (32%)	128,630 (60%)	0 (0%)	
Not applicable	221,248 (55%)	33,008 (15%)	188,240 (100%)	
Follow-up Time[3] (year)	13.4 (12.6, 14.3)	13.5 (12.7, 14.3)	13.4 (12.5, 14.2)	<0.001

[1]Mean, (SD); [2]n (%); [3]Median (IQR)
[4]Welch Two Sample t-test; Pearson's Chi-squared test; Wilcoxon rank sum test
Significance level set at alpha = 0.05
MET: Metabolic Equivalent Task; BMI: Body Mass Index; SBP: Systolic Blood Pressure

Supplementary Table 3 Likelihood Ratio Tests comparing Cox models with and without interaction terms between MHCs and sex in relation to cancer incidence

Interaction term	LRT[1]	df	p-value
Depression	6.021	1	0.0141*
Anxiety	2.319	1	0.1278
Bipolar disorder	5.836	1	0.0157*
SZ	1.203	1	0.2728
PTS	0.005	1	0.9445

[1]Likelihood Ratio Tests
Each comparison tests whether including an interaction term between the specified mental health condition and sex significantly improves model fit. All models are adjusted for age, ethnicity, deprivation, smoking status, alcohol intake, physical activity, sleep duration, processed meat intake, BMI, diabetes, and systolic blood pressure.

Supplementary Table 4 Sensitivity analysis for unmeasured confounding showing the hazard ratios and corresponding E-Values for MHCs and cancer risk

Condition/Site	HR (95% CI)	E-Value (Estimate)[1]	E-Value (95% CI Limit)
	Overall Cancer Risk		
Depression	1.13 (1.08, 1.18)	1.51	1.37
Anxiety	1.15 (1.09, 1.21)	1.57	1.40
Bipolar	1.22 (1.05, 1.43)	1.75	1.28
PTSD	1.38 (0.92, 2.06)	2.10	1.00
Schiz	1.08 (0.92, 1.26)	1.36	1.00
	Site-Specific Cancer Risk		
Depression			
Blood	1.34 (1.20, 1.50)	2.02	1.70
Breast	0.82 (0.73, 0.92)	1.75	1.39
Colorectal	1.08 (0.94, 1.25)	1.38	1.00
Liver	1.85 (1.40, 2.45)	3.10	2.14
Lung	1.62 (1.43, 1.84)	2.62	2.21
Ovarian	1.04 (0.78, 1.41)	1.26	1.00
Prostate	1.12 (0.98, 1.28)	1.49	1.00
Uterine	0.71 (0.53, 0.96)	2.15	1.25
Anxiety			
Blood	1.39 (1.22, 1.58)	2.12	1.74
Breast	0.85 (0.75, 0.98)	1.62	1.16
Colorectal	1.01 (0.84, 1.21)	1.11	1.00
Liver	2.01 (1.43, 2.81)	3.43	2.22
Lung	1.55 (1.32, 1.82)	2.48	1.98
Ovarian	1.08 (0.77, 1.52)	1.38	1.00
Prostate	1.20 (1.03, 1.40)	1.70	1.22
Uterine	0.89 (0.64, 1.24)	1.50	1.00

[1]The E-value is the minimum strength of association an unmeasured confounder would need with both the exposure and outcome to move the HR to the null.

Supplementary Table 5 Baseline characteristics of participants included in the analytic sample (n=402,255) and those excluded due to missing covariate data (n=79,143)

Characteristic	Overall<br>N = 481,398	Excluded due to missing data<br>N = 79,143	Included in analysis<br>N = 402,255	p-value[4]
Age[1] (years)	56 (8)	56 (8)	56 (8)	<0.001
sex[2]				<0.001
Female	260,488 (54%)	46,473 (59%)	214,015 (53%)	
Male	220,895 (46%)	32,655 (41%)	188,240 (47%)	
Ethnicity[2]				<0.001
White	452,303 (94%)	71,456 (93%)	380,847 (95%)	
Black	7,855 (1.6%)	1,620 (2.1%)	6,235 (1.6%)	
Chinese	1,538 (0.3%)	261 (0.3%)	1,277 (0.3%)	
Mixed	2,869 (0.6%)	509 (0.7%)	2,360 (0.6%)	
South Asian	9,687 (2.0%)	1,712 (2.2%)	7,975 (2.0%)	
Any other	4,436 (0.9%)	875 (1.1%)	3,561 (0.9%)	
Deprivation[1]	-1.29 (3.10)	-0.88 (3.33)	-1.37 (3.04)	<0.001
Smoking Status[2]				<0.001
Current	51,106 (11%)	9,654 (13%)	41,452 (10%)	
Never	262,794 (55%)	42,965 (56%)	219,829 (55%)	
Previous	164,675 (34%)	23,701 (31%)	140,974 (35%)	
Alcohol[3] (units/week)	11 (2, 23)	9 (2, 20)	11 (3, 23)	<0.001
Sleep Duration[1] (hour)	7.15 (1.09)	7.12 (1.16)	7.16 (1.08)	<0.001
Total physical activity (MET-min/week)[1]	2,401 (2,442)	2,254 (2,456)	2,422 (2,439)	<0.001
Processed Meat Consumption[1]				<0.001
Never	44,774 (9.3%)	7,227 (9.4%)	37,547 (9.3%)	
Less than once a week	145,621 (30%)	23,964 (31%)	121,657 (30%)	
Once a week	139,838 (29%)	22,503 (29%)	117,335 (29%)	
2-5 times a week	129,890 (27%)	20,223 (26%)	109,667 (27%)	
5-6 times a week	15,128 (3.2%)	2,341 (3.0%)	12,787 (3.2%)	
once or more daily	3,968 (0.8%)	706 (0.9%)	3,262 (0.8%)	
BMI[1] (kg/m^2)	27.4 (4.8)	28.1 (5.3)	27.3 (4.7)	<0.001
Self-reported Diabetes[2]	22,351 (4.6%)	4,345 (5.5%)	18,006 (4.5%)	<0.001
SBP[1] (mmHg)	138 (19)	138 (19)	138 (19)	<0.001
Menopausal Status[2]				<0.001
No	63,149 (13%)	10,772 (14%)	52,377 (13%)	
Yes	155,757 (32%)	27,127 (34%)	128,630 (32%)	
Not applicable/ unknown	262,492 (55%)	41,244 (52%)	221,248 (55%)	
Mental health condition diagnosis[2]				<0.001
Diagnosed	48,843 (10%)	9,539 (12%)	39,304 (9.8%)	
Undiagnosed	432,555 (90%)	69,604 (88%)	362,951 (90%)	
Follow-up Time (year)[3]	13.5 (12.6, 14.3)	14.0 (12.8, 14.6)	13.4 (12.6, 14.3)	<0.001

[1]Mean, (SD); [2]n (%); [3]Median (IQR)
[4]Welch Two Sample t-test; Pearson's Chi-squared test; Wilcoxon rank sum test
Significance level set at alpha = 0.05
MET: Metabolic Equivalent Task; BMI: Body Mass Index; SBP: Systolic Blood Pressure

Supplementary figures

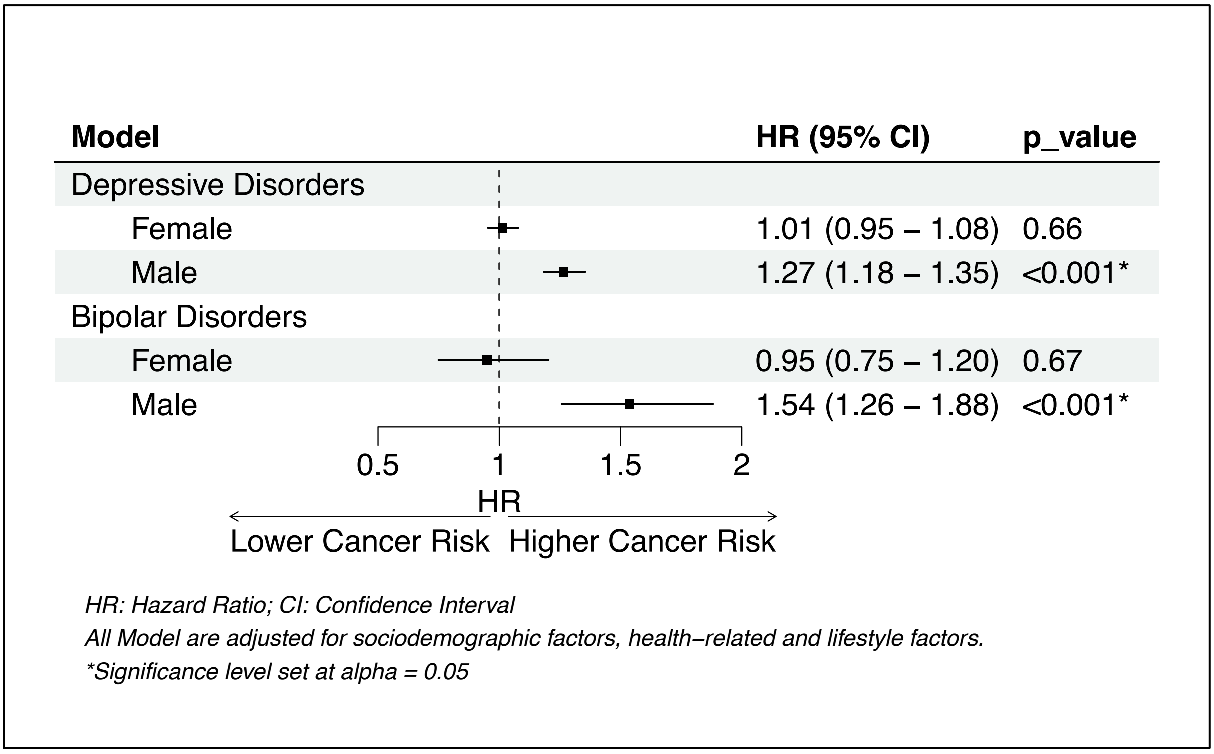

Supplementary Figure 1 Forest plot of Cox models for depressive disorders and bipolar disorders and overall cancer stratified by sex

Sensitivity Analysis

Model	Number of Events	HR (95% CI)	p_value
Overall Cancer	61948	1.03 (1.02 – 1.04)	<0.001*
Breast Cancer	9166	1.02 (1.00 – 1.04)	0.11
Ovarian Cancer	1150	1.00 (0.94 – 1.06)	0.89
Uterine Cancer	1422	0.96 (0.91 – 1.02)	0.19
Prostate Cancer	10350	1.04 (1.02 – 1.06)	<0.001*
Lung Cancer	4314	1.11 (1.08 – 1.14)	<0.001*
Blood Cancer	8790	1.07 (1.05 – 1.09)	<0.001*
Colorectal Cancer	6150	0.99 (0.97 – 1.02)	0.67
Liver Cancer	887	1.10 (1.04 – 1.18)	0.002*

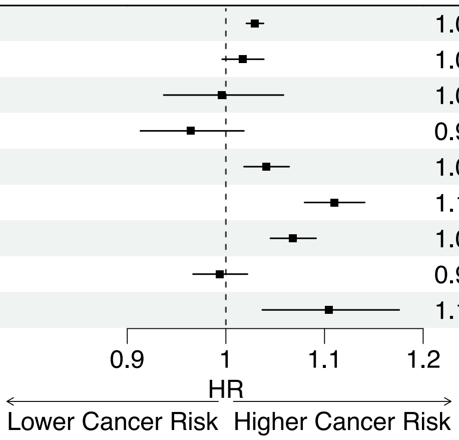

HR: Hazard Ratio; CI: Confidence Interval;
The standardised 4–item Patient Health Questionnaire (PHQ–4) scores represented the main independent variable across all models; All Model are adjusted for sociodemographic, health–related, and lifestyle factors.
**Significance level set at alpha = 0.05*

Supplementary Figure 2 Forest plot of the sensitivity analysis models using PHQ-4
